# Comparative evaluation of ethyl acetate and n‐Hexane extracts of *Cannabis sativa* L. leaves for muscle function restoration after peripheral nerve lesion

**DOI:** 10.1002/fsn3.3255

**Published:** 2023-02-06

**Authors:** Javeria Maqbool, Haseeb Anwar, Azhar Rasul, Ali Imran, Malik Saadullah, Shoaib Ahmad Malik, Asghar Shabbir, Rabia Akram, Faiqa Sajid, Shamaila Zafar, Suman Saeed, Muhammad Numan Akram, Fakhar Islam, Ghulam Hussain, Saiful Islam

**Affiliations:** ^1^ Neurochemicalbiology and Genetics Laboratory (NGL), Department of Physiology, Faculty of Life Sciences Government College University Faisalabad Pakistan; ^2^ Laboratorie of Neuroimmunologia, Department of Physiology and Pharmacology Sapienza University Rome Italy; ^3^ Department of Zoology, Faculty of Life Sciences Government College University Faisalabad Pakistan; ^4^ Department of Food Sciences Government College University Faisalabad Pakistan; ^5^ Department of Pharmaceutical Chemistry, Government College University Faisalabad Pakistan; ^6^ Department of Biochemistry, Sargodha Medical College University of Sargodha Sargodha Pakistan; ^7^ Department of Biosciences COMSATS University Islamabad Pakistan; ^8^ Department of Neurology, Allied Hospital Faisalabad Medical University Faisalabad Pakistan; ^9^ Institute of Nutrition and Food Science University of Dhaka Dhaka Bangladesh

**Keywords:** *Cannabis sativa* L., ethyl acetate, functional recovery, natural compounds, n‐Hexane, oxidative stress, peripheral nerve injury

## Abstract

Peripheral nerve injuries are one of those complex medical conditions for which a highly effective first‐line treatment is currently missing. The use of natural compound as medicines to treat various disorders has a long history. Our previous research explored that crude *Cannabis sativa* L. accelerated the recovery of sensorimotor functions following nerve injury. The purpose of the current study was to investigate the effects of n‐Hexane and ethyl acetate extracts of *C. sativa* L. leaves on the muscle function restoration in a mouse model after sciatic nerve injury. For this purpose, albino mice (*n* = 18) were equally divided into control and two treatment groups. The control group was fed on a plain diet while treatment groups were given a diet having n‐Hexane (treatment 1) and ethyl acetate (treatment 2) extracts of *C. sativa* L. (10 mg/kg body weight), respectively. The hot plate test (M = 15.61, SD = 2.61, *p* = .001), grip strength (M = 68.32, SD = 3.22, *p* < .001), and sciatic functional index (SFI) (M = 11.59, SD = 6.54, *p* = .012) assessment indicated significant amelioration in treatment 1 as compared to treatment 2 group. Furthermore, muscle fiber cross‐sectional area revealed a noticeable improvement (M = 182,319, SD = 35.80, *p* = .013) in treatment 1 while muscle mass ratio of Gastrocnemius (M = 0.64, SD = 0.08, *p* = .427) and Tibialis anterior (M = 0.57, SD = 0.04, *p* = .209) indicated nonsignificant change. A prominent increase in total antioxidant capacity (TAC) (M = 3.76, SD = 0.38, *p* < .001) and momentous decrease in total oxidant status (TOS) (M = 11.28, SD = 5.71, *p* < .001) along with blood glucose level indicated significant difference (M = 105.5, SD = 9.12, *p* < 0.001) in treatment 1 group. These results suggest that treatment 1 has the ability to speed up functional recovery after a peripheral nerve lesion. Further research is necessary, nevertheless, to better understand the extract's actual curative properties and the mechanisms that improve functional restoration.

## Graphical abstract

Neuroprotective and therapeutic role of c. sativa extract.

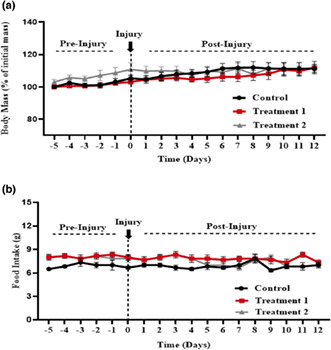

## 1 BACKGROUND

Peripheral nerve injuries (PNIs) fall among the most pivotal issues regarding health status because of their higher prevalence. Such injuries gradually interfere with motor function and result in the loss of sensation in the affected body part (Kouyoumdjian et al., 2017). The PNIs create a hindrance in the brain's communication with the muscles or target organs. Ultimately, depending upon the type, site, and severity, these injuries affect the performance, mobility, perception, sensations of skin, and joints. Most often they result in a life‐long disability of the affected individual (Bray & Huggett, 2016; Noble et al., 1998). In this aspect, different therapeutic interventions and medicinal agents have been developed and practiced; however, these efforts have been constrained because of poor functional outcomes. All of these therapy options merely work to slow down the deterioration of cell bodies (Zafar et al., 2020, 2021). Finding successful treatment options for PNIs involves focusing on the method for maintaining neuromuscular connections, which permits reinnervation of muscles after prolonged denervation of muscles and minimizes damage to the cell body (Akram et al., 2022; Goldfarb & Gelberman, 2005). In particular, phytochemicals are being investigated to identify a more effective alternative for the treatment of PNI.

*Cannabis sativa* L. (*C. sativa* L.), a member of the family Cannabaceae, is commonly known as marijuana or hemp and cultivated in humid and tropical regions worldwide. It has been used as an anti‐inflammatory, analgesic, sedative, hypnotic, and hallucinogenic agent (Wang et al., 2014). It has a variety of cannabinoids (Aizpurua‐Olaizola et al., 2017). It exhibits neuroprotective, neuro‐antioxidative, antitumor, and anti‐inflammatory effects due to the presence of tetrahydrocannabinol (THC) and cannabidiol (CBD) (Andre et al., 2016). Surprisingly, CBD is remarkably effective at decreasing neuropathic pain by reducing inflammation that leads to the regeneration of the sciatic nerve (Costa et al., 2007). THC, and CBD work together to improve muscle function in vivo by reducing inflammation and restoring functioning autophagy. By increasing intracellular calcium concentration mainly through transient receptor potential (TRPV1) activation, an effect that undergoes fast desensitization, CBD stimulated the differentiation of murine C2C12 myoblast cells into myotubes. Not only CBD but also THC stimulated myotube development in primary satellite cells and myoblasts. They reversed the loss of locomotor function, decreased inflammation, and increased autophagy in mdx mice (Iannotti et al., 2019).

We have previously reported that supplementation of a crude *C. sativa* L. leaves speeds up the functional recovery in a nerve injury model. We moved forward to investigate the effects of *C. sativa* L. extracts (leaves) in n‐Hexane and ethyl acetate on the recovery of muscle function following peripheral nerve lesion.

## 2 MATERIALS AND METHODS

### 2.1 Animals

The study was approved by the institutional animal care and ethics committee. This experiment was carried out on the albino mice (*Mus musculus*), arranged by the animal housing facility of the Department of Physiology, Government College University Faisalabad (GCUF). The mice used in this study were weighed between 25 and 30 g and 8‐ to 10‐weeks old. They were housed in individual plastic rodent cages that held one mouse each, at a temperature of ±26°C, ambient humidity of 41%–59%, an unlimited supply of food and water and a 12‐h light and 12‐h dark cycle that was managed by designing appropriate rooms (Kamran et al., 2020; Rasul et al., 2019).

### 2.2 Plants' material preparation and supplementation

The Department of Botany at GCUF made the identification of the *C. sativa* L. leaves (268‐bot‐20), which were obtained from peripheral areas of Faisalabad, Pakistan. The leaves were shade dried, ground into fine powder and soaked in n‐Hexane and ethyl acetate solvents for 10 days. Following this, they were filtered using filter paper and placed in rotary apparatus for evaporation. The respective extract mixed with diet was administered to the experimental groups from the day of nerve crush till the end of the experiment.

### 2.3 Sciatic nerve compression injury and study design

The sciatic nerve crush was induced in all mice following the acclimatization period of five to 6 days. The desired part of the skin was shaved smoothly before inducing nerve crush. We used a mixture of ketamine (70‐mg/kg body weight) and xylazine (5‐mg/kg body weight) to prepare the anesthesia and injected this mixture via the intraperitoneal route to the mice. After being given anesthesia, the right leg's mid‐thigh region was exposed, and sciatic nerve was crushed mechanically by using a fine pair of forceps with a constant force of 12 s. Afterward, two to three stitches were used to close the skin wounds and pyodine was applied to the sutured site to avoid any infection. The hind paw with the sciatic nerve crush was taken as the ipsilateral paw and the opposite hind paw was taken as the contralateral paw or control (Halter et al., 2010; Hussain et al., 2013; Junxiong et al., 2013). All animals (*n* = 18) were equally divided into three groups as follows: normal chow (control), n‐Hexane *C. sativa* L. extract (treatment 1), and ethyl acetate *C. sativa* L. extract (treatment 2). The treatment groups received diets containing n‐Hexane and ethyl acetate extracts of *C. sativa* L. (10‐mg/kg body weight), respectively, whereas the control group received a routine diet. At the end of study, the mice were euthanized by injecting xylazine (5‐mg/kg body weight) and ketamine (70‐mg/kg body weight). After decapitation, blood samples were collected and tissues were harvested (Imran et al., 2019; Razzaq et al., 2020).

### 2.4 Behavioral tests

#### 2.4.1 Sciatic functional index (SFI)

The sciatic functional index (SFI) is an innovative and reliable parameter used to evaluate the motor function restoration following the sciatic nerve crush (Junxiong et al., 2013). To perform this test, the hind paws of the mouse were painted with nontoxic blue ink, and the mouse was allowed to walk on a wooden track (7 cm × 50 cm) with a white chart on its floor. The most clearer and readable four to five footprints were collected for each mice. Then, these footprints were calculated by using the given formula:

$$\begin{array}{c} \mathrm{SFI}=\left(-38.3\times -\frac{\mathrm{EPL}-\mathrm{NPL}}{\mathrm{NPL}}\right)+\left(109.5\times \frac{\mathrm{ETS}-\mathrm{NTS}}{\mathrm{NTS}}\right)\\ \;+\left(13.3\times \frac{\mathrm{EIT}-\mathrm{NIT}}{\mathrm{NIT}}\right)-8.8 \end{array}$$

Print Length (PL) = Measurement of distance from the tip of third toe to heel, Intermediate toe spread (IT) = distance between the second to fourth toe, Toe spread (TS) = Toe spread is the distance between first and fifth toe. NTS, NPL, and NIT denote the normal limbs, whereas ETS, EPL, and EIT denote the experimental paw (Sajid et al., 2021).

#### 2.4.2 Muscle grip strength

This technique was used to assess the muscle strength of mice in vivo by using the ability of mice to clamp a horizontal grid or metal bar of grip strength meter (Bioseb, Chaville, France). When the mouse was placed across a metallic grid, the inadvertent rearward movement was spontaneously observed. A total of three readings were taken for both hind limbs of the mice that is, ipsi‐ and contralateral to the lesion (Hussain et al., 2013).

#### 2.4.3 Hot plate test

It was used to measure the sensory functional recovery of the hind limbs of the mice. Before the actual testing, the mouse was introduced to the nonfunctioning hot plate (SCILOGEX MS7‐H550‐S LED digital 7 × 7 Hotplate stirrer) for 1 min (Haas et al., 2010). After that, they were allowed to stand with their operated hind paw in contact with the device set at a temperature of 56 ± 2°C until they showed any response. This time was taken as the hot plate latency (HPL) (Yu et al., 2008). To record the latency period, a stopwatch was used and considered as a withdrawal reflex (WRL). The mice were removed after 30 s in case of no response and the latency period was recorded as 30 s. Total three readings were taken with a difference of 2 min for each mouse (Mene et al., 2002).

### 2.5 Biochemical tests

#### 2.5.1 Total antioxidant capacity (TAC)

Antioxidants defend the body from oxidative stress and engage in a battle with free radicals (oxidant species). Free radicals are reactive single or multiple atoms or groups having an unpaired electron in a shell. Their excessive production can result in a variety of clinical diseases, including cellular degeneration. The Erel (2004) technique was used to measure the amount of total antioxidants in serum samples of all mice (Erel, 2004).

#### 2.5.2 Total oxidant status (TOS)

Total oxidant status (TOS) refers to the presence of oxidant species in a living system. The body generates these oxidant species as a result of different metabolic pathways. This test depends on the oxidation of o‐Dianisidine ferrous ion into ferric ion since oxidants are present in the biological sample. In the ensuing acidic environment, xylenol orange forms a compound with ferric ions, which adds color. The spectrophotometer (Biolab‐310) was used to detect the color intensity (Erel, 2004; Wu et al., 2017). It is measured in μmol H_2_O_2_ Equiv./L.

#### 2.5.3 Measurement of random blood glucose

Throughout the experiment, the random blood glucose of every mouse was measured in order to determine the glucose contribution to the pathogenesis of PNI. In the experiment, the glucose level was checked twice: first before the sciatic nerve was mechanically crushed, and again after it had been crushed. A digital glucometer (Accu‐chek) was employed to measure blood sugar using the technique of earlier study (Ayala et al., 2010; Brăslaşu et al., 2007).

### 2.6 Muscle weight

At the end of the experiment, the Gastrocnemius and Tibialis anterior of both the ipsi‐ and contralateral hind limbs were harvested and weighed to determine the degree of muscular atrophy (Wu et al., 2015).

### 2.7 Morphometric analysis

The Tibialis anterior muscles of mice were taken out surgically after the dissection and were fixed for 24–48 h in 10% neutral buffer formalin. The tissue was embedded in the paraffin; a 5‐μm section was taken from the rotary microtome and was stained with Hematoxylin and Eosin (H&E). Following this, the cross‐sectional area was quantified by using the IMAGE J software version 1.52.

### 2.8 Statistical analysis

Graph Pad Prism, version 8.0 was used to analyze the data. The results were expressed as mean ± SEM, and ANOVA was used to compare the means. A value of *p* < .05 was considered significant.

## 3 RESULTS

### 3.1 Effect of treatment on body mass and diet intake

**FIGURE 1:**
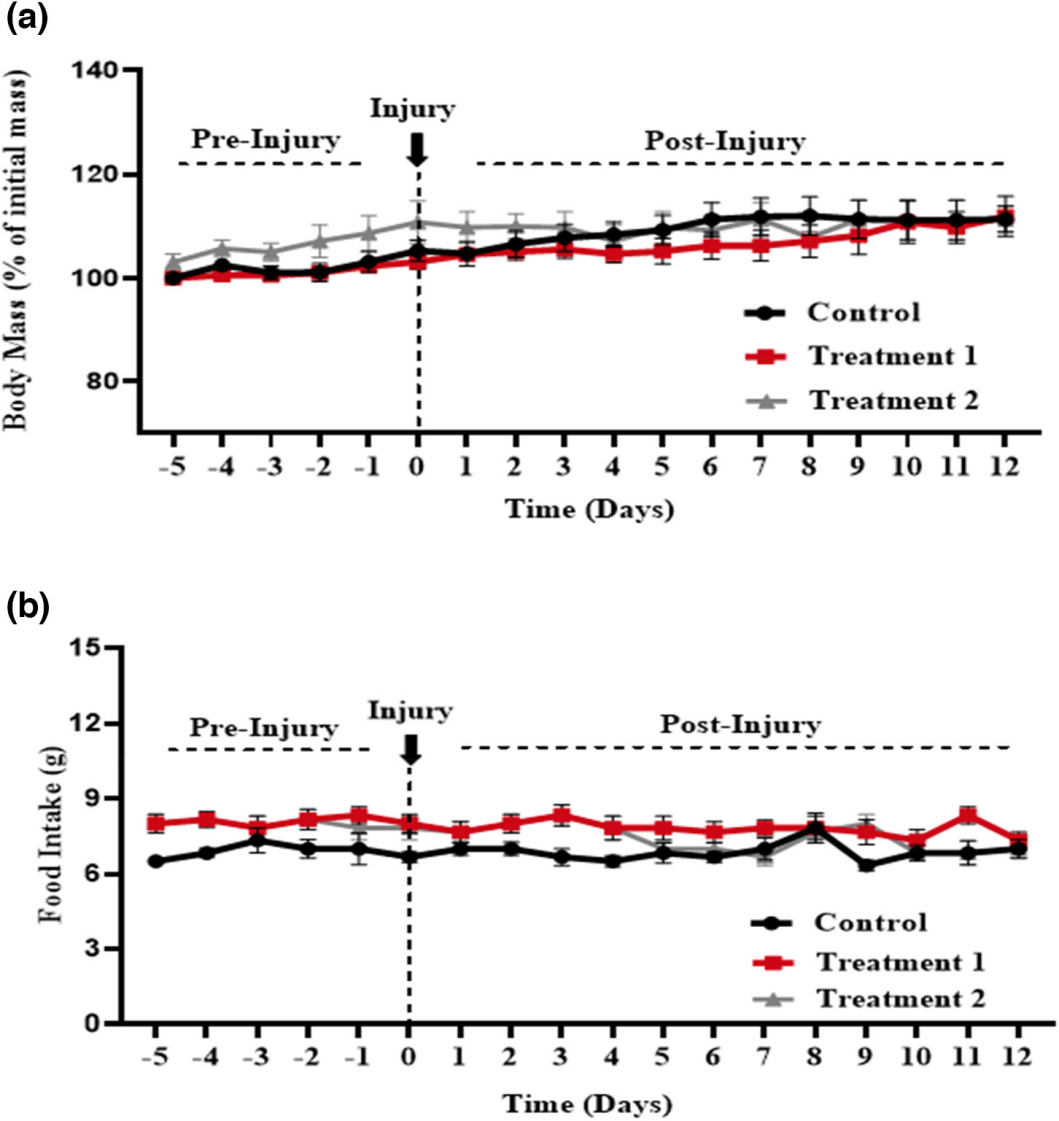
Effect of treatment on body mass and diet intake. Two‐way ANOVA showed a nonsignificant effect of treatments on body mass (*p* = .473) and diet intake (*p* = .550).

The body mass and diet intake were measured in all groups daily during the entire period of experimentation from the day of acclimatization to the end of the trial. It was found that neither the bodyweight nor diet intake was affected by the addition of *C. sativa* L. n‐Hexane and ethyl acetate extracts. The statistical study showed that there is a nonsignificant change among all the groups in body mass and diet intake (*p* > .05) (Figure 1a,b).

### 3.2 Effect of treatment on motor and sensory function recovery

**FIGURE 2:**
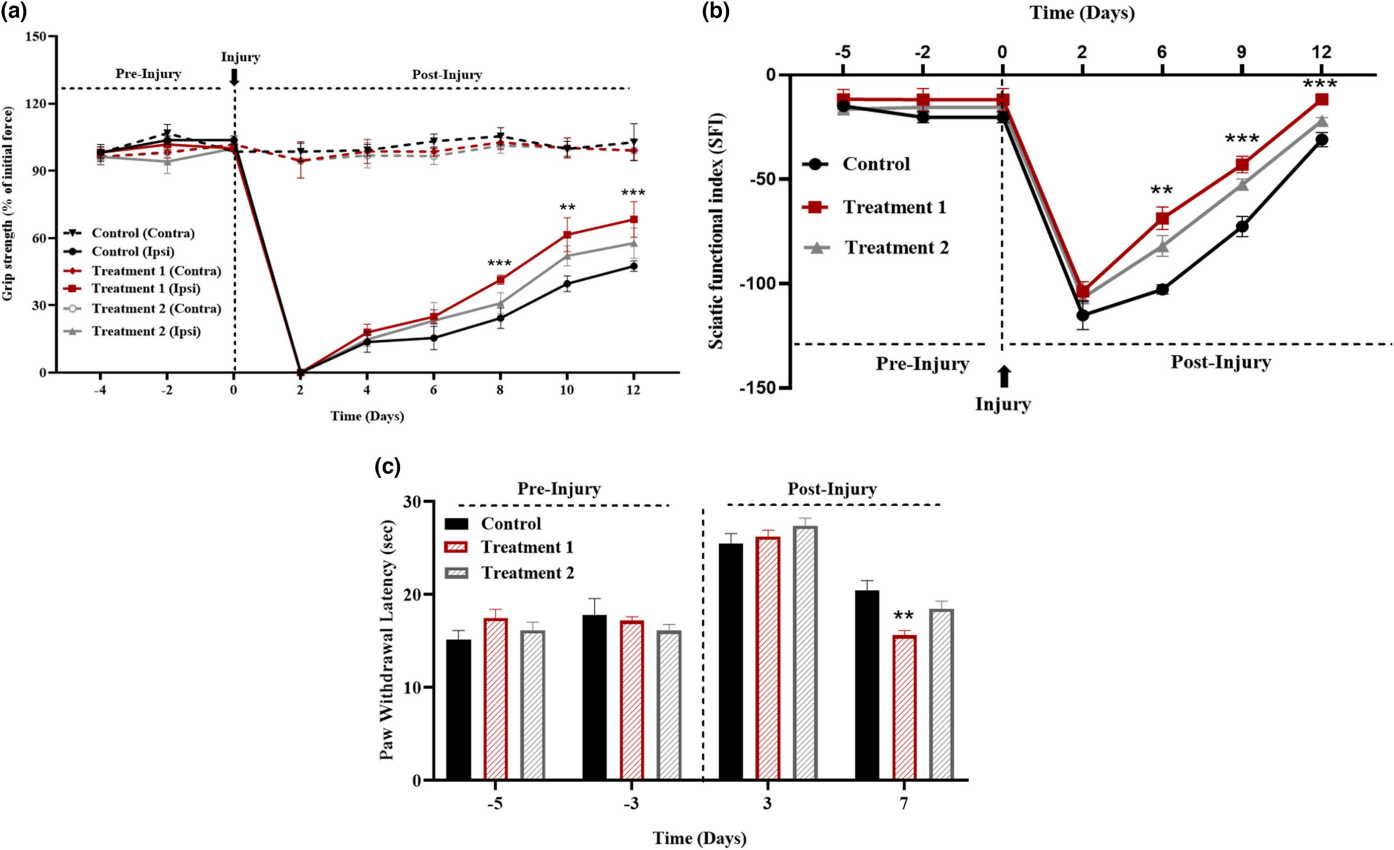
Effect of treatment on motor and sensory function recovery. Muscle grip strength (*p* < .001), SFI (*p* = 0.012), and paw withdrawal latency (*p* = .001) indicated a significant improvement after the treatment in treatment 1 group as compared to treatment 2 and control groups.

We discovered that treatment 1 resulted in an earlier return of motor function when compared to treatment 2 (Figure 2a,b). Grip strength and SFI were assessed at different time points. Both muscle grip strength (*p* < .001) and SFI (*p* = .012) measurements of the recovery of motor function are depicted in the graph as having the same tendency. All groups experienced less sensation right away after the nerve damage. On day 7 (postinjury), there was a substantial increase in paw withdrawal potential (*p* = .001), which indicated that treatment group 1's sensory function recovered more quickly (Figure 2c).

### 3.3 Effect of treatment on oxidative stress and blood glucose levels

**FIGURE 3:**
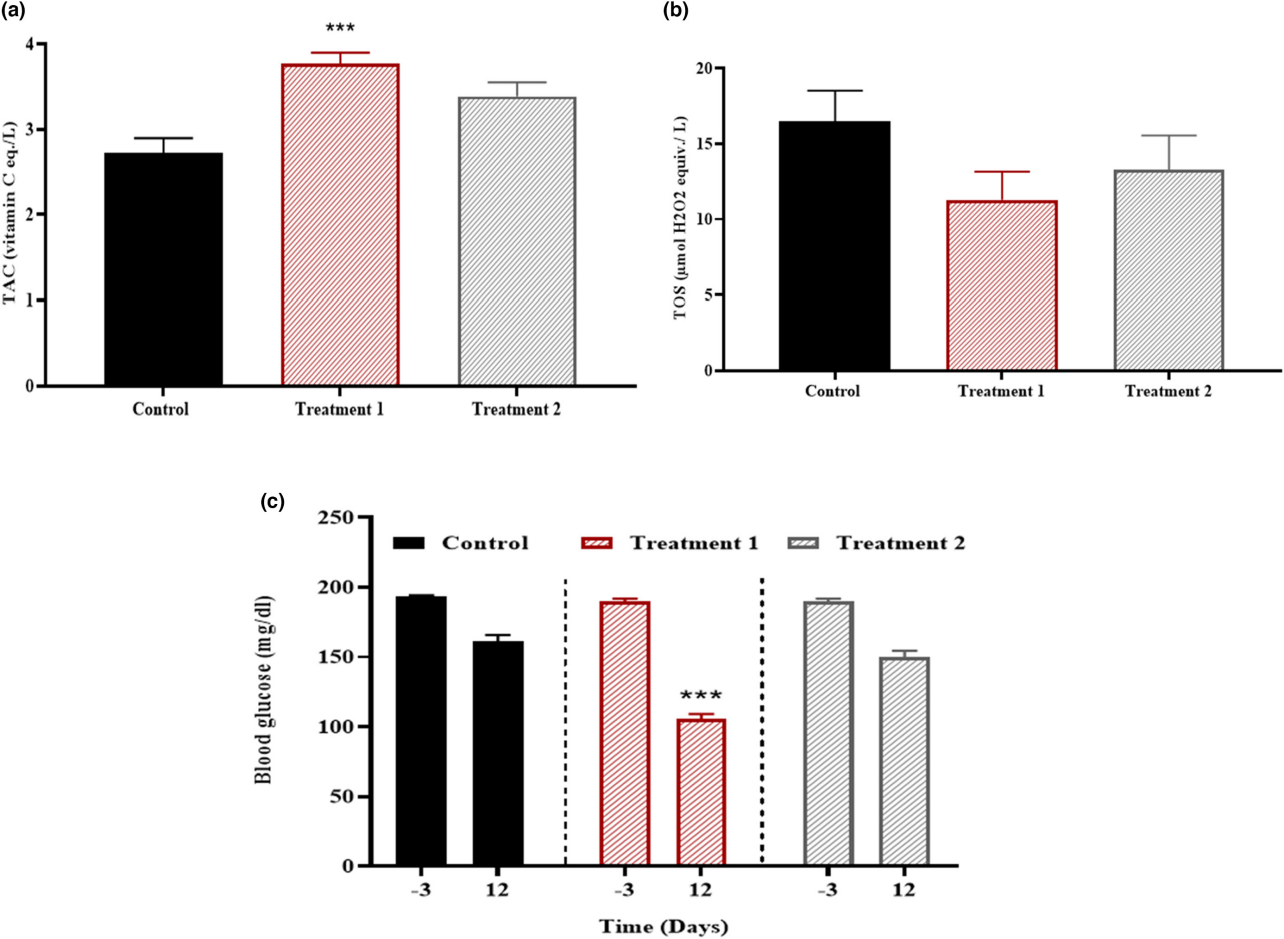
Effect of treatment on oxidative stress and blood glucose. Total antioxidant capacity (TAC) was significantly improved in treatment 1 (***p < .001) group. In contrast, TOS depicted a nonsignificant difference (*p* = .233) in all groups, and blood glucose level indicated a significant change (*p* < .001) in treatment 1 group.

The oxidative stress (TAC, TOS) was measured at the end of trial in each of three groups. A highly significant increase in TAC values (*p* < .001) and a noticeable decrease in TOS (*p* < .001) was observed in treatment 1 as compared to treatment 2 and control group (Figure 3a,b). The blood glucose (*p* < .001) was recorded before persuading the injury and then 12‐days postinjury. Compared to treatment 2, there was a significant drop in glucose levels in treatment 1 (Figure 3c).

### 3.4 Effect of treatment on muscle mass

**FIGURE 4:**
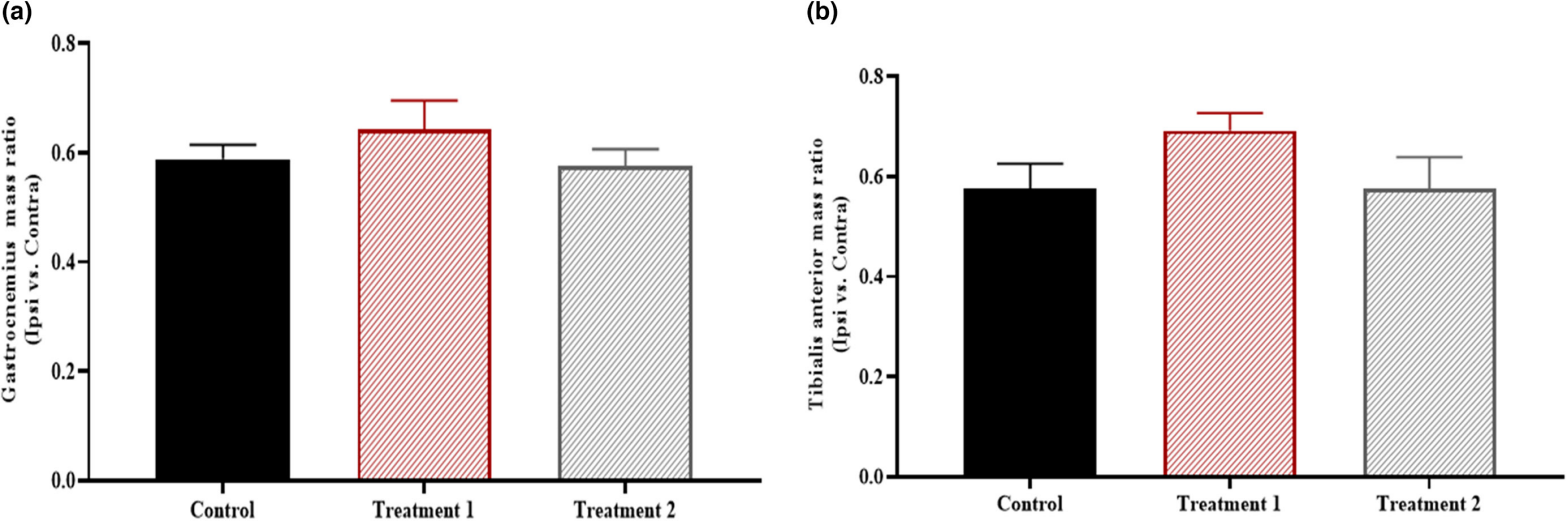
Effect of treatment on the muscle mass ratio. The mass ratio of Gastrocnemius (*p* = .427) and Tibialis anterior muscles showed a nonsignificant difference (*p* = .209) in all groups.

In the treated groups, there was a slight rise in Gastrocnemius (*p* = .427) and Tibialis anterior (*p* = .209) mass, but data were not statistically significant (Figure 4a,b).

### 3.5 Effect of treatment on muscle fibers morphology

**FIGURE 5:**
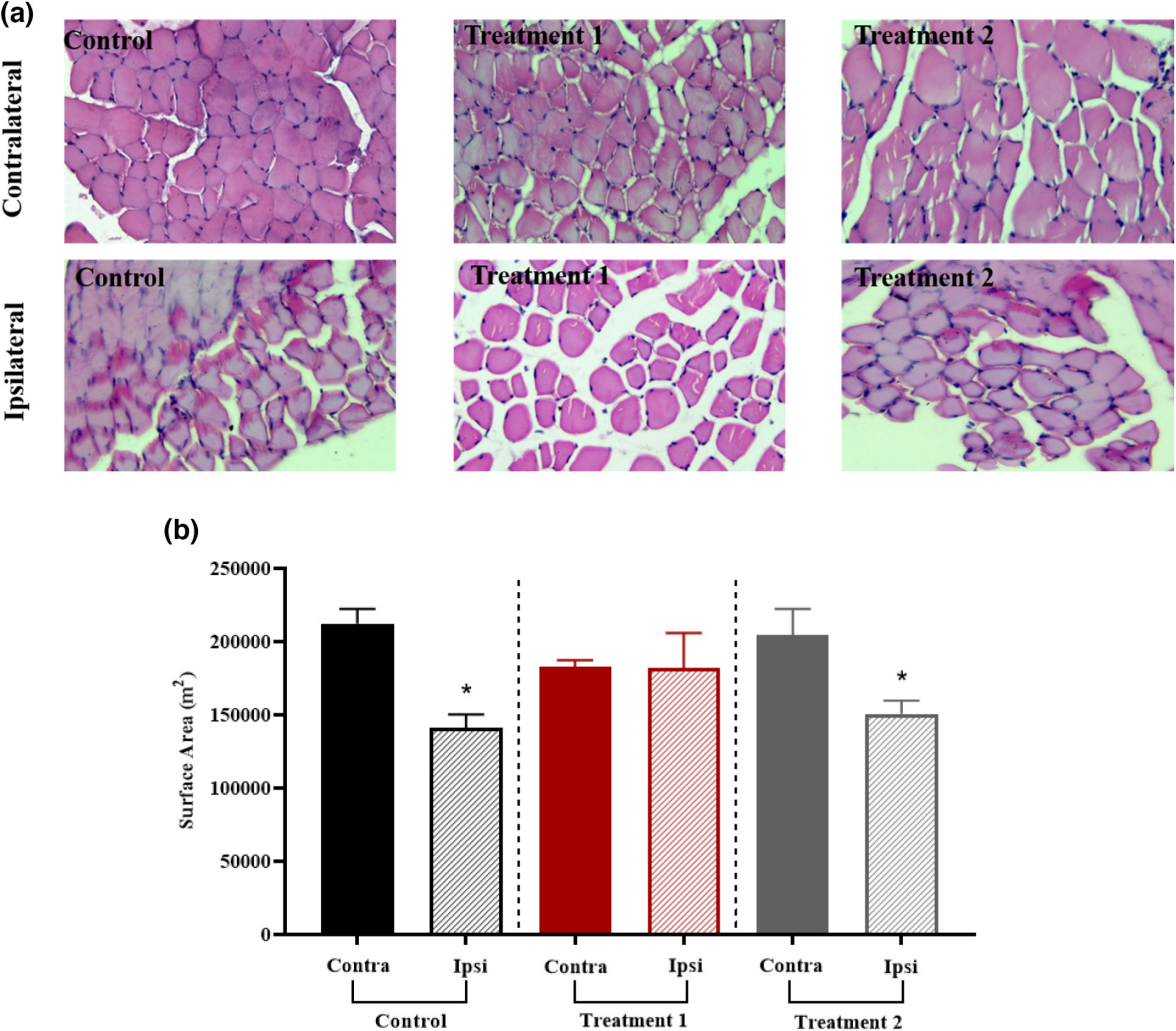
Effect of treatment on the muscle fibers morphology. (a) Representative photographs of the Tibialis anterior muscle cross‐section were viewed under a compound microscope; H&E stained images taken at 40×. (b) Muscle fibers morphology showed a remarkable improvement (*p = .013) in treatment 1 group. In contrast, a nonsignificant difference (*p* > .999) was noted in treatment 2 and control group animals.

The presence of dystrophy is shown by the considerable difference in the cross‐sectional area of the ipsilateral and contralateral muscles in the control group, as well as in treatment 2 group. However, in treatment 1 group, there was no statistically significant difference between the cross‐sectional areas of the ipsilateral and contralateral muscles, demonstrating that treatment 1 was effective in promoting axonal regeneration (Figure 5).

## 4 DISCUSSION

It is commonly known that *C. sativa* L. has medicinal properties and it can be used as medicine. Although there is a wealth of data on its antioxidant, antidepressant, neuroprotective, and anxiolytic effects, its role in improving functional recovery following PNI has never been dissected out. We have already assessed whether *C. sativa* L. leaves have the potential to regenerate and reinnervate the functional retrieval against sciatic nerve crush injury (Aziz et al., 2019). The findings of this study suggested that this plant may be useful due to both its neuroprotective and neuroregenerative effects in restoring sensorimotor function lost as a result of nerve compression injury. We assessed crude form of this plant, though. In order to further explore the potential effects of *C. sativa* L. leaves in two distinct solvents, such as n‐Hexane and ethyl acetate, on accelerating functional recovery, this study is an extension of our prior research.

Body mass and food consumption were monitored every day at the same time, and the results were nonsignificant. This indicates that neither treatment 1 nor treatment 2 ingestion altered the animals' eating pattern. The hot plate test was used to evaluate sensory functions, and the SFI and measurement of grip strength were used to evaluate motor functional recovery. According to the results of all these measures, treatment 1 shown a considerable improvement in functional recovery in terms of hot plate (*p* = .001), grip strength (*p* < .001), and SFI (*p* = .012) in comparison to treatment 2 and the control group. The early sensorimotor function recovery points to the presence of treatment's neuroregenerative potential (Aziz et al., 2019).

The sciatic nerve directly innervates the Gastrocnemius and Tibialis anterior muscles. These muscles are used for walking, running, and the inward and outward flexion of the foot. Thus, as the sciatic nerve is squeezed, these two muscles get denervated, and as the muscle continues to lose nerve conduction, its bulk declines, which eventually results in functional loss (Glass, 2005). If the neuronal loss to the innervated muscles continues for a long time, it can cause everlasting muscular dystrophy that would be impossible to recover (Hussain et al., 2020). So, a quick recovery is essential to prevent muscle degeneration from becoming permanent. Therefore, we also assessed the muscle mass ratio between the Gastrocnemius (*p* = .427) and the Tibialis anterior (*p* = .209) muscles. Although the statistical findings were determined to be nonsignificant, the graphical illustration of the muscle mass ratio (Ipsi vs. Contra) demonstrated that the value of the muscle ratio for both muscles in treatment group 1 was nearly equal. Any damage to a nerve prevents electrical signals from reaching the target muscle. In the extended absence of such impulses, muscle fibers atrophy. The health state of muscle fibers is typically determined by their tiny cross‐sectional area and irregular shape. In this context, each group's ipsilateral and contralateral Tibialis anterior muscles underwent morphometric examination. According to statistical analysis, the ipsilateral and contralateral muscles in the control and treatment 2 group showed a significant difference (*p = .013), whereas the ipsilateral and contralateral muscles in treatment 1 showed no significant difference (*p* > .999). It shows how significantly *C. sativa* L. influences the rate of axonal regrowth.

One of the main issues at the injury site is oxidative stress; free radicals produced there worsen tissue damage and slow down the process of neuroregeneration. Conversely, reduced oxidative stress or increased antioxidants improve functional recovery after PNI. Oxidative stress is one of the primary causes of neuronal damage by inducing mitochondrial malfunction and apoptosis, which causes neuroinflammation and demyelination (Areti et al., 2014). Here, we discovered that the relatively lower TOS level (*p* < .001) and increased TAC level (*p* < .001) in treatment 1 group, suggesting the strong antioxidant potential of *C. sativa* L. leaves. The findings of this study convincingly corroborate past findings and validate functional recovery following nerve damage (Aziz et al., 2019).

It is widely known that elevated blood glucose levels produce pathological symptoms at the site of nerve damage, disrupt the normal glucose metabolism, and mimic a number of pathological symptoms. In this case, blood sugar levels were measured both before and after the injury in order to evaluate the therapeutic potential of *C. sativa* L. extracts in controlling blood sugar levels as reported in earlier studies (Ayala et al., 2010; Brăslaşu et al., 2007). After a few days of treatment 1, we observed a considerable drop in blood sugar levels (*p* < .001), which supports its hypoglycemic impact when compared to control and treatment 2. It is conceivable that several phytoconstituents in treatment 1 alter the cellular process by which glucose is metabolized, ultimately improving the pace of nerve regeneration. The current findings support our earlier research that helped to clarify *C. sativa* L.'s possible antidiabetic function (Aziz et al., 2019).

## 5 CONCLUSION

In a nutshell, the results of this investigation demonstrate that n‐Hexane *C. sativa* L. leaves extract has the ability to hasten the recovery of functions following a compression damage to the sciatic nerve. Even though these results are very encouraging and validating our previously reported data, however, more in‐depth research is advised to investigate the key participants in the supported recovery process. Future research on *C. sativa* L. may reveal it to be a cutting‐edge medicinal agent for the regeneration of peripheral nerves in cases of traumatic injury.

## FUNDING INFORMATION

The authors declare that no funds, grants, or other support were received during the preparation of this manuscript.

## CONFLICT OF INTEREST STATEMENT

The authors have no relevant financial or nonfinancial interests to disclose.

## ETHICAL APPROVAL

The use of the mouse model for the current project was approved by the Institutional Review Board (IRB No. 629), Government College University, Faisalabad, Pakistan.

## CONSENT TO PARTICIPATE

All the co‐authors are willing to participate in this manuscript.

## CONSENT FOR PUBLICATION

All authors are willing for publication of this manuscript.

## Data Availability

Even though adequate data has been given, however, all authors declare that if more data required then the data will be provided on request basis.
